# Quality of Life in Wilson’s Disease: A Systematic Literature Review

**DOI:** 10.36469/jheor.2021.29987

**Published:** 2021-12-08

**Authors:** Chakrapani Balijepalli, Kevin Yan, Lakshmi Gullapalli, Stephane Barakat, Helene Chevrou-Severac, Eric Druyts

**Affiliations:** 1 Pharmalytics Group; 2 Alexion Pharmaceuticals; 3 Alexion Pharma GmBH

**Keywords:** quality of life, health-related quality of life, Wilson’s disease, hepatolenticular degeneration, genetic disorder

## Abstract

**Background:** Wilson’s disease (WD) is a rare inherited genetic disorder characterized by the progressive accumulation of copper in the brain, liver, and other major organ systems. To date, there have been no comprehensive studies synthesizing evidence pertaining to the quality of life (QOL) in WD. **Objective:** We conducted a systematic literature review to identify and synthesize the evidence on QOL in patients with WD. **Methods:** To address this gap in the literature, we conducted a systematic literature review in MEDLINE and EMBASE to identify observational studies and clinical trials reporting QOL outcomes among people living with WD. **Results:** A total of 442 publications were identified, 41 publications were eligible for full-text screening, and 7 articles, representing 7 studies, met all inclusion criteria. QOL questionnaires used across studies included the 12-Item Short Form Health Survey Questionnaire (version 1) (SF-12) (n=2), the 36-Item Short Form Health Survey Questionnaire (version 1) (SF-36) (n=3), Global Assessment Scale (GAS) (n=1), and World Health Organization QOL brief questionnaire (WHO-QOL-BREF) (n=1). Overall, the pattern in QOL from most studies demonstrated a worse QOL in WD patients compared with the general population, a deterioration in QOL for patients presenting with neurologic symptoms, and more frequent psychiatric symptoms compared with the ones with hepatic symptoms. **Discussion:** Although our understanding of the underlying pathophysiology of WD has advanced, and novel therapeutics are on the horizon, our understanding of how WD affects overall QOL remains limited. Evidence from this review demonstrates the substantial heterogeneity in reporting outcomes pertaining to the QOL associated with WD. These differences may be attributable to the fact that QOL is not typically assessed and the lack of a standardized method for assessing QOL in WD. **Conclusion:** This review demonstrates a need for more up-to-date studies with larger sample sizes to further evaluate QOL in patients with WD. The study also demonstrates the need for a WD-specific instrument to measure the QOL in WD patients.

## INTRODUCTION

Wilson’s disease (WD) is a rare inherited genetic disorder characterized by the overaccumulation of copper in the brain, liver, and other major organ systems.^1^ WD is caused by a homozygous or compound heterozygous mutation in the *ATP7B* gene that encodes a membrane transporter for copper excretion.^1–3^ Progressive copper accumulation has a negative impact on multiple organs and tissues, including hepatic (acute liver failure, active hepatitis, cirrhosis), neurologic (Parkinson-like symptoms), psychiatric (depression, psychosis), ocular (Kayser-Fleischer corneal rings, cataracts), renal (renal tubular acidosis, urolithiasis, proximal/distal tubular dysfunction, proteinuria), and muscular (rhabdomyolysis, osteoporosis) complications.^4^

A recent review reported the prevalence of WD to be approximately 1.5-3.5 per 100,000 individuals across the United States, Europe, and Asia.^5,6^ WD affects an equal number of both males and females and is found in all ethnic groups and races.^7^ Approximately 1 in 90 individuals is a heterozygous carrier of the *ATP7B* disease gene.^8^ WD typically presents in teens and young adults; however, it can become symptomatic in persons at any age.^9,10^ WD is generally diagnosed within 6 months to 3 years of initial symptom presentation.^11,12^ Persons with WD experiencing neurologic complications have a longer symptom duration before diagnosis compared with those with hepatic complications.^12^ Predominantly, WD clinically presents as hepatic (vomiting, ascites, fluid buildup in legs, jaundice, and itchiness) or neurological (tremor, muscle stiffness, speech impairment, anxiety, personality changes, and auditory or visual hallucinations) manifestations. However, in young children, the majority of WD cases are asymptomatic and are usually discovered on familial screening or abnormal liver function test results.^4,13,14^

Given that patients often present with hepatic, neurologic, and psychiatric manifestations, management of WD should involve a multispecialty approach. In WD, no new therapeutic options have been introduced in over 50 years.

Currently, copper chelators (penicillamine, dimercaprol, trientine, and dimercaptopropane sulfonate) and/or drugs that prevent copper absorption in the gastrointestinal tract (zinc salts) are the mainstay of treatment.^15^ Current therapies do not always improve neurological symptoms and may even cause paradoxical worsening. Further, they may not be well tolerated or difficult for patients to adhere to because they must be taken while fasting multiple times per day.^12^ Apart from the anticopper treatments, in WD patients with severe neurological symptoms, such as tremor, dystonia, parkinsonism, and chorea, other treatments such as anticholinergics, benzodiazepines, dopamine receptor antagonists, dopamine-depleting drugs, and carbamazepine/oxcarbamazepine can be used. If these other drugs fail, neurosurgical treatments can be employed.^16^ Potential future therapies include tetrathiomolybdate, which binds tightly to copper and forms a tripartite complex with albumin; an oral copper-protein binding agent; and a gene therapy that corrects the defective *ATP7B* transporter in WD.^17,18^ Previous studies have shown that WD patients who receive adequate care have a good prognosis. The life expectancy in patients who are diagnosed early and treated adequately is similar to that of the general population.^19–21^ Moreover, treatment not only prolongs life but also helps these individuals experience improved quality of life (QOL).^22^

There is a lack of comprehensive evaluation across the mental and physical QOL domains in individuals with WD and between its associated disease manifestations. Prior research has qualitatively reported QOL in individual studies; however, an exhaustive literature search and synthesis has not been conducted.^23^ We conducted an exhaustive systematic literature review and synthesis of studies reporting QOL outcomes among people with WD.

## METHODS

### Literature Searches and Eligibility Criteria

A systematic literature search was conducted in MEDLINE and EMBASE for all literature published from 1979 for EMBASE and 1946 for MEDLINE up to July 24, 2021 (**Tables S1-S2)**. Conferences from the past 2 years, including the International Society for Pharmacoeconomics and Outcomes Research (ISPOR 2018 and 2019), European Conference on Rare Diseases & Orphan Products (ECRD 2018), American Association for the Study of Liver Diseases (AASLD 2018 and 2019), and Asian Pacific Digestive Disease Week (APDW 2018 and 2019), were also searched. Hand searches of practice guidelines, national and international orphan disease organizations, and the bibliographies of any relevant articles were reviewed for any studies potentially not captured by the databases. Studies were included based on the Population, Intervention, Comparator, Outcomes, and Study (PICOS) design criteria, as defined in **Table S3**. In summary, eligible studies included observational studies or clinical trials reporting QOL or health-related QOL outcomes reported by patients, caregivers, or clinicians in individuals diagnosed with WD.

### Data Screening and Extraction

All abstracts were screened according to the PICOS criteria. Relevant abstracts were screened again by viewing the full-text study publication to determine a final inclusion status as outlined by the PICOS criteria. Data extracted from these studies included study characteristics (study design, intervention, geographic location, study duration, and period), participant characteristics (age, sex, age at diagnosis, treatment regimen, disease severity, comorbidities), and patient- and clinician-reported outcomes (PRO/ClinRO) (eg, QOL and health-related QOL).

Study screening and data extraction were performed independently by two reviewers. These individuals compared their completed work to identify any discrepancies and resolve these through consensus, including a third individual if needed. The PRISMA checklist was used to ensure completeness of all reported items **(Table S4)**.^24^

### Evidence Synthesis

Due to the heterogeneity in the study objectives, meta-analysis methods could not be applied. Instead, only a qualitative synthesis was performed on the design of the studies and outcomes used.

### Quality of Life Instruments

The SF-36 and SF-12 are generic self-report questionnaires with 36 and 12 questions, respectively, to evaluate an individual’s health status or QOL. Both SF-36 and SF-12 assess the health status of a patient in 8 domains: physical functioning, physical role, bodily pain, general health, vitality, social functioning, emotional role, and mental health. Scores from these sections are transformed into a 0-100 scale with higher scores meaning lesser disability. The WHOQOL-BREF, an instrument derived from data collected using WHOQOL-100, produces scores for one question from each facet relating to QOL (ie, physical, psychological, social relationships, and environment) and 2 questions from the overall QOL and general health facets, for a total of 26 questions scored from 1 to 5. Global Assessment Score (GAS) is a two-part clinician-reported evaluation tool. Tier 1 scores global disability across four domains: liver, cognition, behavior, motor, and osseomuscular, with an ascending six-point scale of 0-6, where lower scores correspond to better health and higher score corresponds to worse health. A further assessment of Tier 2 domains includes a multidimensional scale analysis of neurological dysfunction with 14 items, including Wilson’s facies, cognition and behavior, movement disorders, bulbar symptoms, posture and gait impairment, and Kayser-Fleischer rings. These items are rated on an ascending 5-point scale in which lower scores correspond to better health and higher scores correspond to worse health.

## RESULTS

**Figure 1.**
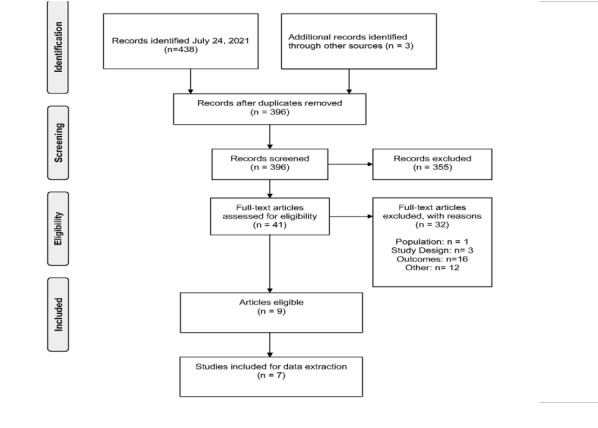
PRISMA Diagram

The process to identify studies for inclusion is summarized in the PRISMA flow diagram in Figure 1. The bibliographic search identified a total of 438 publications via MEDLINE and EMBASE. Three studies were further identified through scanning the gray literature. Of the 442 studies identified, 41 publications were eligible for full-text screening. Although a total of 9 articles met all inclusion criteria, only 7 full-text articles were included, as the other 2 were not full-text publications.

The majority of studies (n=4) were conducted in Europe (Germany, Italy, Serbia, United Kingdom), 2 studies were conducted in India, 1 study was conducted in both the United States and Europe. Three studies were cross-sectional, two were prospective, one study was retrospective, and one was a case-control study; no clinical trial was identified. When we applied the National Institutes of Health quality assessment tools, of the 6 studies included in this analysis, 4 were considered good quality, and 3 were considered fair quality. The mean age of individuals in the included studies ranged from 16.5 to 42.4 years. Of the 6 included studies, 1 study compared QOL in participants with WD to individuals without the condition, and the remaining 5 studies evaluated QOL in a single group of individuals with WD (Table 1). Four studies reported baseline characteristics of individuals receiving treatment for their condition. Across these studies, individuals had current or prior treatment experience with penicillamine, trientine, and/or zinc. Individuals with predominant neurologic and hepatic complications associated with WD were represented across the included studies.

**Table 1.** Study Design Characteristics of Included Studies

**Study**	**Region/** **Country**	**Study Type**	**Study Design**	**Age**	**Study Period**	**Total WD, n**	**Eligibility Criteria**	**Study Objective**	**QOL/HRQOL** **Measurement Used**
Schaefer et al (2016)^25^	Germany	Full-text	Retrospective-cross sectional study	≥18	--	68	All patients included had confirmed diagnosis of WD, attended our tertiary WD care center, and were 18 years of age or older. Patients receiving antipsychotic or antidepressant co-medication and patients who underwent liver transplantation for WD were excluded from the study.	Measure the QOL in WD patients and compare QOL across gender and treatment regimen	SF-36
Carta et al (2012)^26^	Italy	Full-text	Case-control study	--	--	23	Patients diagnosed and treated for WD	Compare and evaluate the impairment of quality of life between individuals with WD and individuals without. Moreover, assess the relationship between mood disorders and impaired quality of life in WD through	SF-12
Svetel et al (2011)^27^	Serbia	Full-text	Cross-sectional study	--	--	60	Patients with WD recruited from the WD Clinical Research Program at the University of Belgrade who were clinically stable (no significant clinical change in the preceding 6 months) and had been on standardized therapy for WD for at least 2 years (ie, at the time of investigation, all patients were in a copper-depleted state)	Measure the QOL in WD patients	SF-36
Aggarwal et al (2009)^28^	India	Full-text	Prospective study	All ages	2006-2007	30	--	--	GAS
Komal Kumar et al (2007)^29^	India	Full-text	Retrospective study	>18	--	30	Patients were selected from a large cohort of WD, followed at a tertiary care University teaching hospital, from south India. Inclusion criteria for the study were the definite diagnosis of WD and treatment. Patients who were unable to answer the questionnaire due to behavioral problems, low IQ or other disease related factors per se were excluded.	Measure the QOL in WD patients	WHO-QOL-BREF
Sutcliffe et al (2003)^30^	UK	Full-text	Prospective study	All ages	1988-2000	21	Patients diagnosed with WD and underwent orthotopic liver transplantation	Assess quality of life in a group of individuals diagnosed with WD whom had undergone liver transplantation	SF-36
Camarata et al (2021)^31^	USA and Europe	Full-text	Cross-sectional study	>18	2017-2020	62	Patients diagnosed and treated for WD	To assess the QOL of patients with WD and the relationship between mental and physical health QOL and severity of liver disease, neurological disease, and the most common psychiatric problems in patients with WD: major depressive disorder (MDD) and cognitive impairment.	SF-12

GAS, Global Assessment Scale; HRQOL, health-related quality of life; MINI, MINI International Neuropsychiatric Interview; MOCA, Montreal Cognitive Assessment; PedsQL, Pediatric Quality of Life Inventory; QOL, quality of life; SF-12, Short Form-12 Health Survey Questionnaire; SF-36, Short Form-36 Health Survey Questionnaire; WHO-QOL-BREF, World Health Organization QOL brief questionnaire.

QOL questionnaires used in the included studies comprised the SF-12 (n=2), SF-36 (n=3), WHO-QOL-BREF (n=1), and GAS (n=1). Characteristics among participants enrolled in the included studies are provided in Table 2.

**Table 2.** Characteristics of Individuals With WD Enrolled in Included Studies

**Study**	**Total WD (n)**	**Study Population**	**Age** **mean (SD)**	**Male,** **n (%)**	**Mean Age at Diagnosis** **(SD)**	**Mean Age at Symptom Onset (SD)**	**Duration of Disease (yr)**	**Latency Period in Years (SD)**	**Medical Treatment, n (%)**					**Duration of** **Treatment in Years**	**Hepatic,** **n (%)**	**Neuro-logical,** **n (%)**	**Mixed,** **n (%)**	**NS,** **n (%)**
									**Any**	**PEN**	**TRI**	**Zinc**	**Combo**					
Schaefer et al (2016)^25^	68	All	36.6 (12.9)	29 (43)	18.5 (11)	16.6 (10.6)	—	2.6 (4.9)	68 (100)	36 (53)	16 (24)	13 (19)	3 (4)	—	38 (56)	7 (10)	10 (15)	—
	39	Female	37.9 (3.5)	0	17.3 (11.3)	16.1 (3.9)	—	2.4 (5.1)	39 (100)	17 (44)	12 (31)	9 (23)	1 (2)	—	21 (54)	4 (10)	4 (10)	—
	29	Male	35.2 (12.2)	29 (100)	20.6 (10.6)	17.8 (12.7)	—	3.2 (3.1)	29 (100)	19 (66)	4 (14)	4 (14)	2 (6)	—	17 (59)	3 (10)	6 (21)	—
	37	Pencilamine	—	19 (51)	13.1 (5.3)	—	—	—	37 (100)	37 (100)	0	0	0	12.6 (12.1)	26 (70)	2 (5)	6 (16)	—
	16	Trientine	—	4 (25)	19.6 (9.7)	—	—	—	16 (100)	0	16 (100)	0	0	8.1 (5.6)	7 (44)	2 (12)	2 (12)	—
	13	Zinc	—	4 (31)	24.2 (13.9)	—	—	—	13 (100)	0	0	13 (100)	0	8.6 (4.4)	4 (31)	2 (15)	2 (15)	—
	2	Combo	—	2 (100)	14.4 (1)	—	—	—	2 (100)	0	0	2 (100)	0	12.2	1 (50)	1 (50)	0	—
Carta et al (2012)^26^	23	Cases	42.02 (12.52)	9 (39.1)	30.19^c^ (18.28)	—	—	—	—	—	—	—	—	—	—	—	—	—
	92	Controls	42.35 (10.29)	36 (39.1)	—	—	—	—	—	—	—	—	—	—	—	—	—	—
Svetel et al (2011)^27^	60	All	36.8 (11.0)	36 (60)	—	24 (8.3)	—	22.1 (32.6)a	60 (100)	39 (65)	1 (1.7)	8 (13.3)	12 (20)	11.3 (8.2)	19 (31.7)	41 (68.3)	0	—
Aggarwal et al (2009)^28^	30	All	17 (7-40)^b^	17	—	—	—	—	30 (100)	30 (100)	—	—	—	—	—	—	—	8
Komal Kumar et al (2007)^29^	30	All	27.97 (11.16)	23	20.3 (10.7)	16.8 (7.1)	11.08 (9.31)	—	30 (100)	—	—	—	—	9.2 (6.4)	1	27^d^	2	—
Sutcliffe et al (2003)^30^	21	All	16.5^c^ (8-41)^b^	6	—	—	—	—	—	—	—	—	—	—	21 (100)	—	—	—
Camarata et al (2021)^31^	62	All	41 (30-56)^b^	36 (58.06)	19 (11-25)^b^	—	—	—	—	—	—	—	—	19.5(7-35)^b^	12 (19.67)	50 (80.65)	—	—

Abbreviations: Combo, combination therapy; NS, no symptoms; PEN, penicillamine; TR, trientine.

^a^ Months

^b^ Range

^c^ Median

^d^ Two each had psychiatric, musculoskeletal involvement.

**Table 3.** SF-12/SF-36 Outcomes Assessed in Included Studies

**First Author**	**n**	**Study Group**	**Overall Score (SD)**	**Phys Fxn**	**Phys Role**	**Body Pain**	**Gen'l Hlth**	**Vitality**	**Social Fxn**	**Emot Role**	**Mental Hlth**	**PHC**	**MHC**
**Schaefer (SF-36)^25^**	68	All	—	—	—	—	—	—	—	—	—	—	—
	39	Female	73.0	86.0	81.0	79.0	59.0	54.0	78.0	77.0	67.0	72.0	67.0
	29	Male	81.0	90.0	90.0	88.0	67.0	55.0	84.0	97.0	75.0	78.0	75.0
	38	WD-HEP	77.0	91.0	86.0	81.0	65.0	54.0	81.0	87.0	73.0	75.0	72.0
	7	WD- NEURO	61.0	61.0	68.0	60.0	49.0	51.0	66.0	67.0	64.0	58.0	59.0
	36	PEN	81.0	93.0	92.0	90.0	67.0	57.0	83.0	91.0	75.0	80.0	75.0
	16	TRI	70.0	78.0	78.0	76.0	59.0	49.0	81.0	73.0	68.0	68.0	66.0
	13	Zinc	67.0	81.0	71.0	70.0	54.0	50.0	74.0	79.0	58.0	65.0	63.0
**Carta (SF-12)^26^**	23	Cases	33.76 (9.0)	—	—	—	—	—	—	—	—	—	—
	92	Controls	38.14 (6.4)	—	—	—	—	—	—	—	—	—	—
**Svetel (SF-36)^27^**	60	All	71.1 (24.8)	81.1 (26.8)	68.8 (41.3)	77.1 (31.7)	58.9 (26.1)	62.9 (27.9)	75.4 (29.2)	77.8 (37.7)	67.1 (26.2)	69.7 (25.1)	68.4 (25.1)
	19	WD-HEP	81.5 (13.4)	92.4 (10.3)	90.8 (25.3)	—	—	—	—	—	77.1 (17.0)	80.0 (15.9)	77.7 (15.8)
	41	WD- NEURO	66.3 (27.4)	75.8 (30.4)	58.5 (43.5)	—	—	—	—	—	62.4 (28.5)	65.0 (27.2)	64.1 (27.5)
**Sutcliffe (SF-36)^30^**	18	All	—	—	—	—	—	—	—	—	—	47.0 (11.0)	51 (10.0)
	12	Female	—	—	—	—	—	—	—	—	—	43.0 (12.0)	48.0 (11.0)
	6	Male	—	—	—	—	—	—	—	—	—	54.0 (4.0)	57 (5.0)
	11	Acute LF	—	—	—	—	—	—	—	—	—	46.0 (13.0)	47.0 (10.0)
	5	Chronic LF	—	—	—	—	—	—	—	—	—	44.0 (9.0)	58.0 (5.0)
	2	Subacute LF	—	—	—	—	—	—	—	—	—	55.0 (3.0)	57.0 (5.0)
	5	Major AE	—	—	—	—	—	—	—	—	—	44.0 (12.0)	54.0 (2.0)
	13	No major AE	—	—	—	—	—	—	—	—	—	48.0 (11.0)	50.0 (12.0)
**Camarata (SF-12)^31^**	62	All										55.7 (50- 58.4)	50.1 (41.7- 56.9)
	47	NEURO	—	—	—	—	—	—	—	—	—	55.63 (47.9- 58.38)	50.04 (41.71- 57.28)
	11	No NEURO	—	—	—	—	—	—	—	—	—	56.90 (54.35- 58.6)	52.49 (35.28- 55.22)
**Camarata (SF-12)^31^**	27	Cognitive impairment	—	—	—	—	—	—	—	—	—	53.78 (49.74- 58.38)	50.00 (40.86- 56.92)
	28	No cognitive impairment	—	—	—	—	—	—	—	—	—	57.01 (53.02- 58.49)	51.08 (42.37- 55.06)
	12	Cirrhosis	—	—	—	—	—	—	—	—	—	55.66 (53.58- 57.61)	54.72 (51.23- 58.76)
	26	No cirrhosis	—	—	—	—	—	—	—	—	—	55.48 (47.9- 58.60)	45.96 (39.89- 55.22)
	22	Lifetime MDD	—	—	—	—	—	—	—	—	—	54.03 (44.96- 57.48)	42.85 (36.33- 52.80)
	35	No lifetime MDD	—	—	—	—	—	—	—	—	—	56.31 (53.37- 58.38)	52.65 (44.97- 57.28)
	33	Male	—	—	—	—	—	—	—	—	—	55.63 (53.56- 58.38)	46.52 (36.33- 54.93)
	25	Female	—	—	—	—	—	—	—	—	—	54.59 (44.84- 56.92)	54.59 (44.84- 56.92)

Abbreviations: AE, adverse event; GAS, Global Assessment Scale (GAS); Gen’l Hlth, general health; LF, liver failure; MDD, major depressive disorder; PEN, penicillamine; Phys Fxn, physical function; Phys Role, physical role; TRI, trientine; WD-HEP, Wilson’s disease with predominant hepatic complications; WD-NEURO, Wilson’s disease with predominant neurologic complications; WHO-QOL-BREF, World Health Organization QOL Brief Questionnaire.

### Quality of Life

Among the 7 included studies, 5 used the SF-36/SF-12, 1 used the WHO-QOL-BREF, and 1 used the GAS (Table 3).

**Studies with SF-36 assessment:** Schaefer et al (2016) included 68 subjects (57% female) with WD and compared SF- 36 scores across clinical presentation, treatment, and biologic sex. The mean age of the patients was 36.6 years.^25^ The proportion of patients with hepatic, mixed, and neuropsychiatric manifestations were 56%, 15%, and 10%, respectively. Among these patients, 53% received D-penicillamine, 24% trientine, 19% zinc, and 4% combination treatments. Patients undergoing psychiatric treatment or liver transplantation were excluded. The overall SF-36 score was significantly lower in subjects presenting with neuropsychiatric manifestation compared with hepatic (77 vs 61; *P* < 0.005). Compared with a mixed manifestation of WD (both hepatic and neuropsychiatric symptoms present), subjects presenting with hepatic complications only had higher mean SF-36 scores (77 vs 61 for the total score; 72 vs 59 for mental health; 91 vs 61 for physical function), however, these differences were not statistically significant. Penicillamine-treated patients had the highest QOL score compared with those receiving other interventions (penicillamine vs trientine, 81 vs 70; penicillamine vs zinc, 81 vs 67). The authors mentioned that the analysis of potential differences between treatment with zinc and trientine showed no significant differences in QOL using SF-36 scores. Female subjects were found to have significantly lower overall QOL compared with males (73 vs 81); scores were lower across all dimensions of the SF-36.

Across domains, the difference in scores was significant for the sum of the mental health scores, mental health, and the emotional role (sum of physical health, 72 vs 78; sum of mental health, 67 vs 75). For all dimensions of the SF-36, males were shown to experience better QOL compared with females.^25^ Risk of depression was also assessed in this study by the use of the Patient Health Questionnaire-9 and was present in 56% of all patients, although correlation with QOL was not studied.

Svetel et al (2011) reported general SF-36 scores in 60 treated, clinically stable WD patients (ie, no significant clinical change in the preceding 6 months). Subjects were 40% female, with a mean age of 36.8 years.^27^ The proportion of patients with hepatic and neurological manifestations were 31.7% and 68.3%, respectively. Of these patients, 65% were receiving D-penicillamine, 1.7% trientine, 13.3% zinc, and 20% combination treatments. Mean overall SF-36 score was 71.1 (standard deviation [SD]: 24.8). General health was the lowest scored dimension (58.9) and physical function was the highest (81.1)(SD: 26). WD patients with neurologic complications had lower scores across all SF-36 dimensions than patients with hepatic manifestations. Patients with psychiatric symptoms had also a lower QOL than those without such symptoms. Statistically significant differences were found between patients with the neurological form of WD and those with the hepatic form of WD, with lower scores in the former in overall SF-36 score (66.3 vs 81.5, *P*=0.026), physical functioning (75.8 vs 92.4, *P*=0.025), physical role (58.5 vs 90.8, *P*=0.04), mental health (62.4 vs 77.1, *P*=0.043), and physical health (65 vs 80) composite score domains.^27^ The following items were predictive of a poorer QOL in WD patients: time from disease onset to treatment initiation (longer time to treatment was associated with lower QOL), neurological manifestation of WD, lower Minimal Mental State Examination and the 21-item Hamilton Depression Rating Scale scores, and all four domains of the GAS.

Sutcliffe et al (2003) reported QOL in 18 WD patients (75% female; median age, 16.5 years) who had undergone liver transplantation.^30^ The study did not report an overall SF-36 score; however, it did report SF-36 scores for the physical and mental component scores (PCS and MCS) in patients surviving 5 years after transplantation with a functioning graft (mean PCS: 47 [SD: 11]; mean MCS: 51 [SD: 10]. These scores were comparable to age- and sex- matched controls from the general population. The study also noted no significant difference in either PCS or MCS of the SF-36 in relation to sex (male vs female), clinical presentation (acute liver failure vs chronic liver failure), or presence of major adverse events.^30^ The study did not demonstrate an association between early or late major adverse events (eg, reoperation or retransplantation), but a significant correlation was shown between PCS and social functioning.

**Studies with SF-12 assessment:** Carta et al (2012) compared SF-12 scores between 23 persons with WD (60.9% female; mean age: 42 years) and 92 persons without WD (60.9% female; mean age: 42.3 years).^26^ People with WD were found to have lower overall SF-12 scores (mean: 33.76 ± 9.0) compared with those without WD (mean: 38.14 ± 6.4). A subanalysis compared SF-12 scores of persons with WD who also had bipolar disorder and major depressive disorder (MDD). People with WD and bipolar disorder (n=7) experienced diminished QOL (mean: 27.5 ± 7.1). The mean SF-12 score in persons with WD and MDD (n=11) was 34.4 ± 8.2). No statistical significance testing was conducted.^26^

Camarata et al (2021) used the SF-12 to assess the QOL in 62 WD patients treated in the United States and Europe.^31^ The authors observed that WD patients had a lower median mental health score (50.1 vs 55.7) relative to their physical health score. The mean mental and physical scores were similar between the treated WD patients and the general US population. This study also observed that patients with a lifetime diagnosis of MDD had a lower mental health score than patients without a lifetime diagnosis of MDD (42.85 vs 52.65); however, among these patients, physical health scores were not significantly different. This study concluded that mental health score was associated with depression but not cognitive impairment, neurological disease, or liver disease severity. Physical health score was associated with the severity of both neurological and liver disease but not with mental health.

**Studies with GAS assessment:** Aggarwal et al (2009) validated the GAS in 30 WD patients (43.3% female; mean age: 17 years) starting penicillamine therapy.^28^ It was the first publication validating the GAS in WD patients as a means to capture the multisystemic manifestation of the disease and track disease progression and treatment response. Interrater agreement between 2 raters demonstrated reliability, and convergent validity was demonstrated when the GAS domains were compared with other scales capturing the burden of WD. To study responsiveness of the GAS in treated patients, QOL was compared after 3 months between treatment-naïve persons and persons who had received treatment. The Cohen effect size was used to determine the change in QOL (responsiveness of the scale) where the effect size was defined as small (0.2-0.49), moderate (0.5-0.79), and large (≥0.8). Cohen’s effect size for treatment naïve individuals included Tier 1 domains (liver: 0, cognition/behavior: 0.54, motor: 0.78, osseomuscular: 0.19) and treatment experienced (Tier 1 liver: 0, cognition/behavior: 0.45, motor: 0.14, osseomuscular: 0.16). Tier 2 neurologic assessment results for treatment-naïve individuals and treatment-experienced individuals were 0.69 and 0.12, respectively.^27^ This longitudinal follow-up every 3 months over 1.5 years demonstrated that the GAS is particularly sensitive to clinical change among treatment-naïve patients: the GAS showed greater variation between visits among these patients, whereas it was stable for patients treated with penicillamine before the study inclusion. Thus, the GAS was demonstrated to be a reliable scale to measure disability and neurologic assessment in patients.

**Studies with WHO-QOL-BREF assessment:** Komal Kumar et al (2008) used the WHO-QOL-BREF to assess QOL in 30 treated WD patients (23.3% female; mean age: 27.9 ± 11.16 years; mean duration of treatment: 9.2 ± 6.4 years).^29^ The patients were followed regularly for a minimum of 2 years. The study reported QOL results, scored from 1 to 5, by health domain; physical (mean: 3.65, SD: 0.55), psychological (mean: 3.53, SD: 0.75), social relations (mean: 3.93, SD:0.95) and environmental (mean: 3.47, SD: 0.62). All four domains were reported to correlate with each other.^30^ The authors tested the correlation of scores with clinical severity and the QOL of the patients. The clinical severity was captured by the Neurological Symptoms Score (NSS), which ranges from 0 (no disability) to 46 (severe disability). The physical domain of the WHO-QOL-BRED correlated negatively with the NSS (*P*<0.05) and positively with the duration of the treatment (*P*<0.01), demonstrating that more severe disease correlated with greater limitations in physical ability and that longer treatment correlated with better physical ability. None of the other domains of the WHO-QOL-BREF correlated with the NSS, age, or duration of treatment.

## DISCUSSION

We performed a systematic literature review to better understand and synthesize the extent of research performed on assessing the impact of WD on QOL. The majority of the studies evaluated QOL in a single group of participants with WD, whereas the minority compared persons with WD to the general population. Mental and general health were among the QOL dimensions affected most by WD. Overall, in patients with WD (excluding liver transplant recipients), a lower overall QOL score appears to be more closely related to lower scores in the mental health dimensions relative to the physical components. However, the difference appears to be marginal due to the small number of patients included in these studies. Therefore, it is difficult to determine any potential clinical relevance. It can be inferred from the available evidence that patients with WD experience more complications (eg, neurologic symptoms) and, regardless of treatment, have a lower QOL compared with people without WD. Individuals with primary neurologic complications associated with WD appear to have the lowest QOL compared with other forms of WD.

All studies in our review examined multiple dimensions of QOL, including both the psychiatric and physical impact on patients with WD. Evaluation of QOL included the SF-12/SF36 questionnaires (capturing physical function, physical role, body pain, general health, vitality, social functioning, emotional role, mental health), the GAS (global disability and neurological assessment domains), and the WHO-QOL-BREF (physical, psychological, social relations, environmental). Evidence from this review demonstrates the substantial heterogeneity in reporting outcomes pertaining to the QOL associated with WD. Although the GAS was developed to evaluate the overall functioning of a subject during a specified time on a continuum from psychological or psychiatric sickness to health, the GAS adapted for WD additionally includes some neurologic symptoms and the overall impact of the disease on disability. While the GAS is a ClinRO and assesses the impact of the disease on patients’ social life from a health care professional standpoint, the SF-12, SF-36 and the WHO-QOL-BREF are PROs capturing the impact of the health status of a person on their overall QOL. These instruments are called generic PROs, as they capture the impact of the global health status of the person on many dimensions related to QOL and are not specific to any disease. These PRO or ClinRO instruments can capture different dimensions of the impact of the disease on QOL, whether overall QOL or disability, and are not mutually exclusive. A prior systematic review assessed the frequency, QOL, and severity of psychiatric disorders in patients with WD but did not comprehensively report how WD affects overall QOL.^23^ There have been studies that previously investigated WD impact on mental health. For instance, Seniów et al (2003) compared individuals with and without WD and individuals with rheumatoid arthritis using the Hopkins Symptom Check List.^32^ The study demonstrated that patients with WD experienced lower interpersonal sensitivity and anger-hostility compared with healthy controls. Moreover, patients with WD scored significantly lower on retarded depression and phobic anxiety compared with people with rheumatoid arthritis. A trend showed patients with asymptomatic WD scored slightly lower compared with healthy controls in the following attributes: agitated depression, retarded depression, obsessive-compulsive symptoms, anger- hostility, phobic anxiety, and interpersonal sensitivity. However, this trend was not statistically significant due to the small sample size.^32^ Portala et al (2000) assessed 26 treated WD patients using the Comprehensive Psychopathological Rating Scale. Individuals in the study reported signs and symptoms of fatiguability (62%), lack of appropriate emotion (62%), concentration difficulties (62%), observed autonomic disturbances (62%), reduced sleep (54%), and apparent sadness (54%).^33^ These scores indicate the total burden of the psychopathological symptoms in patients with WD, similar to that of patients with moderate to severe depressive disorders.^33^ Although psychiatric symptoms (eg, sadness, anxiety) can greatly influence a person’s overall QOL, it represents only one component of an individual’s perception of QOL; more comprehensive assessments are needed.^34^ As shown by previous studies, psychiatric symptoms are common with WD. Therefore, a multidisciplinary treatment approach considering hepatic, neurological, and psychiatric components is needed. Further, clinical studies should be performed with detailed psychiatric assessment scales, which can help clinicians understand the effectiveness of different treatment options in patients with psychiatric WD.^35^

### Strengths and Limitations

To our knowledge, this is the first systematic review to comprehensively explore and report on dimensions of QOL in people with WD. QOL was reported in the overall population and, when available, further subgroups were explored in the included studies (eg, gender, treatment history, WD subtype). This study also has some limitations, notably, considerable heterogeneity among the included studies. Some studies included persons with predominant neurologic manifestations of WD, whereas others included WD patients with predominant hepatic manifestations. Moreover, it is unclear whether individuals in two of the included studies were or have previously received treatment for WD.^26,30^

One study reported QOL of WD patients within 3 to 139 months after transplantation.^30^ These patients were more likely to experience lower QOL compared with matched controls from the general population. Furthermore, these patients likely had a worse WD prognosis and a more severe form of the disease. QOL outcomes also varied due to the mix of questionnaires used (SF-12, SF-36, GAS, and WHO-QOL-BREF); the heterogeneity in the QOL instruments and the lack of a standard QOL instrument for WD patients makes a comparison of QOL across the studies challenging. Due to the heterogeneity in the study populations and reported QOL outcomes, a meta-analysis could not be conducted.

## CONCLUSIONS

While our understanding of the underlying pathophysiology of WD has advanced and novel therapeutics are on the horizon, our understanding of how WD affects overall QOL remains limited. It is clear from the identified evidence that WD has a negative impact on overall QOL, including physical, mental, emotional, and social functioning. However, there is a dearth of longitudinal evidence assessing the long-term impact of the disease and therapeutics on QOL. Additionally, the available published literature does not seem to differentiate between disease-specific QOL and generic QOL in WD. There is a clear need for further differentiation and consistent measurement of QOL in patients with WD, including the development of QOL instruments that better address the concerns of WD patients.

## Supplementary Material

Online Supplementary Material This supplementary material has been provided by the authors to give readers additional information about their work.
