# Supplementary material for: Quality of Life in Wilson’s Disease: A Systematic Literature Review
Source: J Health Econ Outcomes Res. 2021 Dec 8;8(2):105–13. doi: 10.36469/jheor.2021.29987 (PMC8655222; doi:10.36469/jheor.2021.29987)
Supplement: Online Supplementary Material — This supplementary material has been provided by the authors to give readers additional information about their work. [file jheor_2021_8_2_29987_75961.pdf]

Journal of Health Economics and Outcomes Research

Online Supplementary Material

Balijepalli C, Yan K, Gullapalli L, Barakat S, Chevrou-Severac H, Druyts E. Quality of Life in Wilson's Disease: A Systematic Literature Review. *JHEOR*. 2021:8(2):105-113. doi:10.36469/jheor.2021

Table S1. EMBASE Search
Table S2. MEDLINE Search Strategy
Table S3. PICOS Criteria

This supplementary material has been provided by the authors to give readers additional information about their work.

This is an open-access article distributed under the terms of the Creative Commons Attribution 4.0 International License (CCBY-4.0). View this license's legal deed at http://creativecommons.org/licenses/by/4.0 and legal code at http://creativecommons.org/licenses/by/4.0/legalcode for more information.

Table S1. EMBASE Search Strategy

No.	Search Terms*	Results
1	exp wilsons disease/	10 289
2	(wilsons disease or Wilson disease or Wilson's disease).mp. [mp=title, abstract, heading word, drug trade name, original title, device manufacturer, drug manufacturer, device trade name, keyword, floating subheading word, candidate term word]	11 701
3	hepatolenticular degeneration.mp.	577
4	progressive lenticular degeneration.mp.	14
5	copper storage disease.mp.	38
6	or/1-5	11 741
7	socioeconomics/	145 802
8	exp quality of life/	534 461
9	quality of life.ab,ti,kw.	493 850
10	((instrument or instruments) adj3 quality of life).ab.	4934
11	quality-adjusted life year/	29 060
12	quality adjusted life.ti,ab,kw.	21 977
13	(qaly* or qald* or qale* or qtime* or life year or life years).ti,ab,kw.	36 662
14	disability adjusted life.ti,ab,kw.	4696
15	daly*.ti,ab,kw.	4613
16	(sf36 or sf 36 or short form 36 or shortform 36 or short form36 or shortform36 or sf thirtysix or sfthirtysix or sfthirty six or sf thirty six or shortform thirtysix or shortform thirty six or short form thirtysix or short form thirty six).ti,ab,kw.	44 503
17	(sf6 or sf 6 or short form 6 or shortform 6 or sf six or sfsix or shortform six or short form six or shortform6 or short form6).ti,ab,kw.	2580
18	(sf8 or sf 8 or sf eight or sfeight or shortform 8 or shortform 8 or shortform8 or short form8 or shortform eight or short form eight).ti,ab,kw.	899
19	(sf12 or sf 12 or short form 12 or shortform 12 or short form12 or shortform12 or sf twelve or sftwelve or shortform twelve or short form twelve).ti,ab,kw.	10 355
20	(sf16 or sf 16 or short form 16 or shortform 16 or short form16 or shortform16 or sf sixteen or sfsixteen or shortform sixteen or short form sixteen).ti,ab,kw.	62
21	(sf20 or sf 20 or short form 20 or shortform 20 or short form20 or shortform20 or sf twenty or sftwenty or shortform twenty or short form twenty).ti,ab,kw.	476
22	(hql or hqol or h qol or hrqol or hr qol).ti,ab,kw.	31 654
23	(hye or hyes).ti,ab,kw.	146
24	(health* adj2 year* adj2 equivalent*).ti,ab,kw.	55
25	(pqol or qls).ti,ab,kw.	668
26	(quality of wellbeing or quality of well being or index of wellbeing or index of well being or qwb).ti,ab,kw.	776
27	nottingham health profile*.ti,ab,kw.	1586
28	nottingham health profile/	545
29	sickness impact profile.ti,ab,kw.	1264
30	sickness impact profile/	2351
31	health status indicator/	3177
32	(health adj3 (utilit* or status)).ti,ab,kw.	102 587
33	(utilit* adj3 (valu* or measur* or health or life or estimat* or elicit* or disease or score* or weight)).ti,ab,kw.	21 377
34	(preference* adj3 (valu* or measur* or health or life or estimat* or elicit* or disease or score* or instrument or instruments)).ti,ab,kw.	15 728
35	disutilit*.ti,ab,kw.	1007
36	rosser.ti,ab,kw.	127

Table S1. EMBASE Search Strategy, *cont'd*

No.	Search Terms*	Results
37	willingness to pay.ti,ab,kw.	10 109
38	standard gamble*.ti,ab,kw.	1149
39	(time trade off or time tradeoff).ti,ab,kw.	2115
40	tto.ti,ab,kw.	1837
41	(hui or hui1 or hui2 or hui3).ti,ab,kw.	2533
42	(eq or euroqol or euro qol or eq5d or eq 5d or euroqual or euro qual).ti,ab,kw.	30 146
43	duke health profile.ti,ab,kw.	114
44	functional status questionnaire.ti,ab,kw.	161
45	dartmouth coop functional health assessment*.ti,ab,kw.	13
46	or/7-45	921 852
47	6 and 46	230

* Materials published 1974–July 24, 2021.

Table S2. MEDLINE Search Strategy

No.	Search Terms*	Results
1	exp wilsons disease/	5948
2	(wilsons disease or wilson disease or wilson's disease).mp. [mp=title, abstract, original title, name of substance word, subject heading word, floating sub-heading word, keyword heading word, organism supplementary concept word, protocol supplementary concept word, rare disease supplementary concept word, unique identifier, synonyms]	6162
3	hepatolenticular degeneration.mp.	6028
4	progressive lenticular degeneration.mp.	10
5	copper storage disease.mp.	27
6	or/1-5	7868
7	Value of life/	5749
8	Quality of life/	212 477
9	quality of life.ab,ti,kf.	309 986
10	((instrument or instruments) adj3 quality of life).ab.	3564
11	Quality-Adjusted Life Years/	13 336
12	quality adjusted life.ti,ab,kf.	14 357
13	(qaly* or qald* or qale* or qtime* or life year or life years).ti,ab,kf.	22 930
14	disability adjusted life.ti,ab,kf.	3904
15	daly*.ti,ab,kf.	3524
16	(sf36 or sf 36 or short form 36 or shortform 36 or short form36 or shortform36 or sf thirtysix or sfthirtysix or sfthirty six or sf thirty six or shortform thirtysix or shortform thirty six or short form thirtysix or short form thirty six).ti,ab,kf.	27 323
17	(sf6 or sf 6 or short form 6 or shortform 6 or sf six or sfsix or shortform six or short form six or shortform6 or short form6).ti,ab,kf.	2285
18	(sf8 or sf 8 or sf eight or sfeight or shortform 8 or shortform 8 or shortform8 or short form8 or shortform eight or short form eight).ti,ab,kf.	540
19	(sf12 or sf 12 or short form 12 or shortform 12 or short form12 or shortform12 or sf twelve or sftwelve or shortform twelve or short form twelve).ti,ab,kf.	6436
20	(sf16 or sf 16 or short form 16 or shortform 16 or short form16 or shortform16 or sf sixteen or sfsixteen or shortform sixteen or short form sixteen).ti,ab,kf.	36

No.	Search Terms*	Results
22	(hql or hqol or h qol or hrqol or hr qol).ti,ab,kf.	19 448
23	(hye or hyes).ti,ab,kf.	74
24	(health* adj2 year* adj2 equivalent*).ti,ab,kf.	48
25	(pqol or qls).ti,ab,kf.	408
26	(quality of wellbeing or quality of well being or index of wellbeing or index of well being or qwb).ti,ab,kf.	625
27	nottingham health profile*.ti,ab,kf.	1190
28	sickness impact profile.ti,ab,kf.	1081
29	exp health status indicators/	321 348
30	(health adj3 (utilit* or status)).ti,ab,kf.	78 121
31	(utilit* adj3 (valu* or measur* or health or life or estimat* or elicit* or disease or score* or weight)).ti,ab,kf.	13 312
32	(preference* adj3 (valu* or measur* or health or life or estimat* or elicit* or disease or score* or instrument or instruments)).ti,ab,kf.	11 935
33	disutilit*.ti,ab,kf.	507
34	rosser.ti,ab,kf.	100
35	willingness to pay.ti,ab,kf.	6525
36	standard gamble*.ti,ab,kf.	878
37	(time trade off or time tradeoff).ti,ab,kf.	1487
38	tto.ti,ab,kf.	1162
39	(hui or hui1 or hui2 or hui3).ti,ab,kf.	1660
40	(eq or euroqol or euro qol or eq5d or eq 5d or euroqual or euro qual).ti,ab,kf.	17 897
41	duke health profile.ti,ab,kf.	89
42	functional status questionnaire.ti,ab,kf.	125
43	dartmouth coop functional health assessment*.ti,ab,kf.	13
44	or/7-43	762 039
45	6 and 44	194

* Databases include Ovid MEDLINE® and Epub Ahead of Print, In-Process & Other Non-Indexed Citations, Daily and Versions®, 1946–July 24, 2021.

Table S3. PICOS Criteria

Criteria	Description
Population	Individuals (all ages) diagnosed with Wilson's disease
Interventions	Not restricted
Comparators	Not restricted
Outcomes	Patient/Physician/Caregiver<br>• Generic quality of life<br>• Disease-specific quality of life<br>• Generic health-related quality of life measures<br>• Disease-specific health-related quality of life measures
Study design	• Randomized controlled trials and non-randomized clinical trials<br>• Observational studies reporting health-related quality of life/utility (e.g. controlled before-and-after studies, interrupted time series studies, historically controlled studies, prospective and retrospective cohort studies, case-control studies, cross-sectional studies, controlled and uncontrolled longitudinal studies)
Language	• Studies published in English will be included
